# Pancreaticoduodenectomy in Patients With Coeliac or Superior Mesenteric Artery Stenosis: A Review of the Literature

**DOI:** 10.7759/cureus.62542

**Published:** 2024-06-17

**Authors:** Hossam Nawara, Mohamed Albendary

**Affiliations:** 1 General/Hepato-Pancreato-Biliary (HPB) Surgery, University Hospitals Plymouth NHS Trust, Plymouth, GBR; 2 General Surgery, East Midlands Deanery, Leicester, GBR

**Keywords:** sma stenosis, pancreatic resection, median arcuate ligament syndrome, coeliac artery stenosis, pancreaticoduodenectomy procedure, whipple procedure

## Abstract

Pancreaticoduodenectomy (Whipple’s procedure) is a technically demanding operation performed for malignant and premalignant conditions of the pancreatic head, duodenum and bile duct.

Awareness of the vascular anatomy, variations, and pathology of this area is essential to achieve safe surgery and good outcomes. The operation involves division of the gastroduodenal artery (GDA) which provides communication between the foregut and midgut blood supply. In patients with coeliac or superior mesenteric artery (SMA) stenosis, this can lead to reduced blood supply to the foregut or midgut organs, with consequent severe ischaemic complications leading to significant morbidity and mortality.

Coeliac artery stenosis is caused by median arcuate ligament syndrome (MALS) in the majority of patients with atherosclerosis being the second most common cause. SMA stenosis is much less common and is caused in the majority of cases by atherosclerosis.

A review of preoperative imaging and intraoperative gastroduodenal artery clamp test is important to identify cases that may need additional procedures to preserve the blood supply.

In this paper, we present a literature review for studies reporting patients undergoing Whipple’s operation with concomitant coeliac axis stenosis (CAS) or SMA stenosis. Analysis of causes of stenosis or occlusion, prevalence, risk factors, different management strategies and outcomes was conducted.

## Introduction and background

Pancreaticoduodenectomy (PD) (Kausch-Whipple procedure) is a technically complex and physiologically demanding operation, performed for malignant and premalignant conditions of the head of the pancreas, duodenum, and distal bile duct. The procedure involves dividing the gastroduodenal artery (GDA) from the common hepatic artery (CHA) and the inferior pancreaticoduodenal artery (IPDA) from the superior mesenteric artery (SMA). These arteries form an arcade around the pancreatic head-duodenal complex (pancreaticoduodenal (PD) arcade), which allows collateral blood flow in either direction in cases of coeliac artery or SMA stenosis [1].

Coeliac artery stenosis (CAS) is a relatively common condition ranging between 4-20% in studies [2], however, it remains asymptomatic in most cases due to the presence of rich collateral supply from the PD arcade. When this collateral supply is interrupted in Whipple's procedure, this can lead to ischaemia of the organs supplied by the coeliac artery including liver, stomach, and pancreatic remnant leading to significant morbidity and mortality.

Several case reports and case series have been published describing CA and SMA stenosis in the context of PD, however there is no consensus on the management approach.

In this study, we conducted a literature review for studies on patients undergoing Whipple’s procedure with concomitant coeliac or SMA stenosis, with an analysis of causes, risk factors, management strategies, and outcomes.

## Review

Materials and methods

PubMed search was performed for articles relevant to this topic. We looked for published articles up to December 2023.

We used the following search query: "((coeliac artery stenosis) OR (SMA stenosis) OR (superior mesenteric artery stenosis) OR (celiac artery stenosis) OR (CAS) OR (median arcuate ligament syndrome) OR (MALS)) AND ((Whipple) OR (pancreaticoduodenectomy) OR (pancreatic resection))".

**Figure 1:**
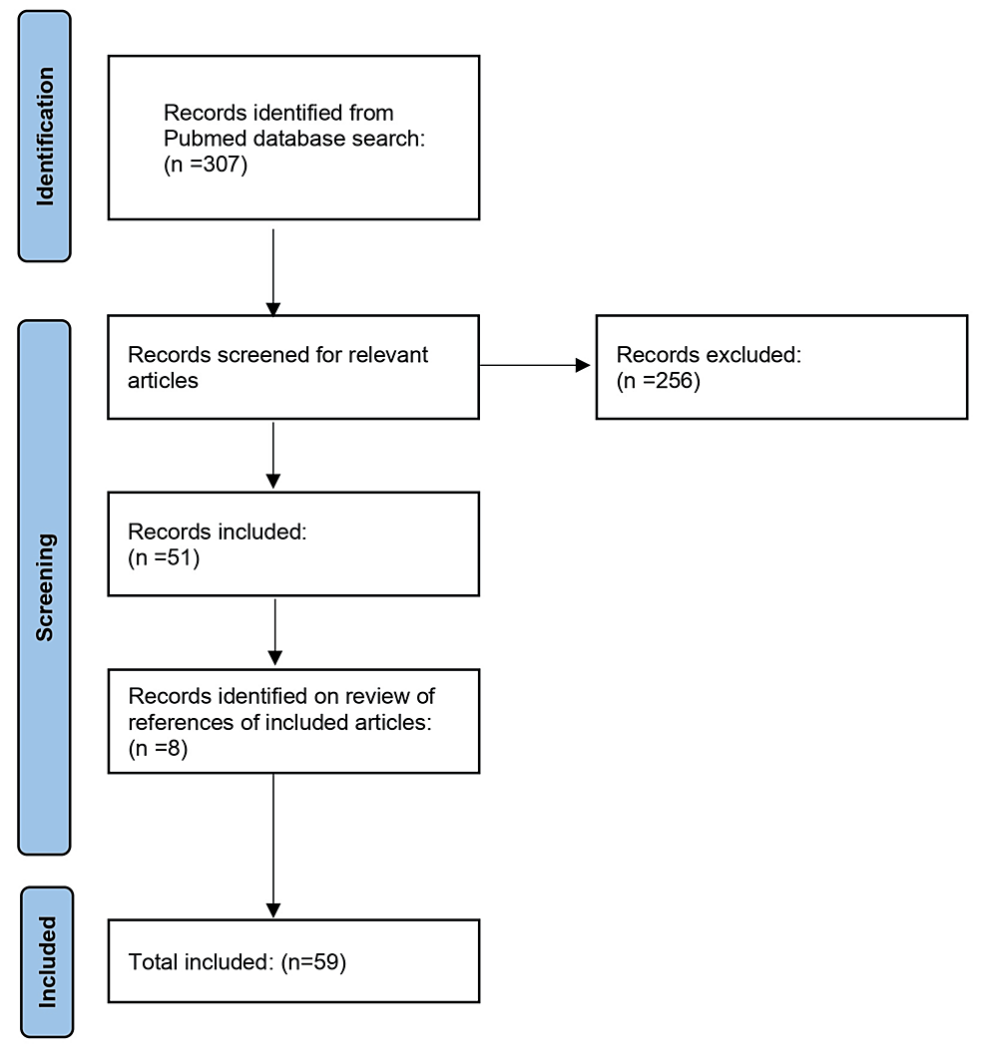
Literature Review Flowchart

The search yielded 307 results. We filtered the search results first by reading through the titles and excluded irrelevant results. Our inclusion criteria are case reports or series of patients with CAS of SMA stenosis and undergoing pancreaticoduodenectomy, or articles discussing this topic. We excluded articles irrelevant to this topic, or case reports where PD was performed in patients with previous surgical history of oesophagectomy or gastrectomy. After screening the search results, we included 51 articles. References of the included articles were checked for relevant studies, and this yielded eight more studies. The total number of included articles was 59 (Figure 1). Articles were reviewed for causes of CAS or SMA stenosis, diagnosis, management and outcomes.

Results

The literature review yielded 136 cases (127 cases of coeliac artery stenosis, and nine cases of SMA stenosis). The cases were reported over a span of years from 1981 to 2023. The average age of those reported was 65 years. Pancreaticoduodenectomy was performed for pancreatic adenocarcinoma in 57 patients, cholangiocarcinoma in 17 patients, ampullary carcinoma in eight patients, duodenal carcinoma in one patient, and for other malignant tumours in five patients. PD was performed in eight patients with benign disease, and in 40 patients, pathology was non-specified. A summary of indications for PD in this study is summarised in Table 1.

**Table 1:** Indications of Pancreaticoduodenectomy

Indication of Pancreaticoduodenectomy	Number of patients
Pancreatic Adenocarcinoma	57
Cholangiocarcinoma	17
Ampullary carcinoma	8
Duodenal Adenocarcinoma	1
Other malignant tumours	5
Benign Pathology	8
Non-Specified	40
Total	136

The cause of coeliac artery stenosis was recorded as median arcuate ligament syndrome (MALS) in 67 patients, atherosclerosis in 12 patients, fibromuscular dysplasia in one patient and non-specified in 47 patients.

Diagnosis of CAS was made preoperatively in 81.9%, intraoperatively in 15.7% and postoperatively in 2.4% of patients.

Management of coeliac artery stenosis varied between studies. A summary of different management modalities is shown in Table 2.

**Table 2:** Summary of different management strategies for coeliac artery stenosis in patients undergoing pancreaticoduodenectomy (PD) MAL: Median Arcuate Ligament; CA: Coeliac Artery.

Definitive treatment	Number of patients
Successful MAL Division	52
Reconstruction (Bypass, anastomosis)	31
Preservation of collaterals	13
Endovascular treatment	15
Retrograde CA stenting	2
Other	5
None	9
Total	127

Division of the median arcuate ligament was performed in 67 patients, and was successful in 52 of them (77%). Of the remaining 15 patients with failed MAL division, vascular reconstruction was performed in five patients, collateral preservation in three patients, endovascular stenting in four patients, open retrograde CAS stenting in one patient, and no treatment in one patient.

The next most common management strategy reported in CAS was vascular reconstruction, which was performed in 31 patients and included variable techniques. Table 3 summarises the various reconstruction techniques performed.

**Table 3:** Different reconstruction methods performed, and number of patients. RHA: Right Hepatic Artery; GDA: Gastroduodenal Artery; MCA: Middle Colic Artery; IPDA: Inferior Pancreaticoduodenal Artery; SMA: Superior Mesenteric Artery; HA: Hepatic Artery; CA: Coeliac Artery.

Reconstruction techniques	Number of patients
Aorta-HA bypass (autologous vein) [3-7]	7
Aorta-HA bypass (PTFE) [8]	2
Aorta-HA bypass (Iliac artery allograft) [9,10]	1
Aorta-CA anastomosis [11]	1
Aorta-CA bypass (autologous vein) [5]	1
Aorta-SA bypass [12]	3
Aorta (supraceliac)-LGA bypass [13]	1
CA angioplasty (vein patch) [14]	1
HA endarterectomy [5]	1
GDA-MCA anastomosis [15,16]	2
GDA-GDA anastomosis [17]	1
GDA-IPDA anastomosis [18,19]	3
GDA-replaced RHA anastomosis (PD and right hepatectomy performed for cholangiocarcinoma [20])	1
Iliac artery-splenic bypass (autologous vein) [21]	2
SMA-HA bypass (autologous vein) [22]	1
Splenic artery-SMA bypass [23]	2
Splenic-IPDA bypass (autologous vein) [24]	1
Total	31

Reconstruction usually involves bypass from the aorta to the hepatic artery, or less commonly to the splenic artery. Autologous vein grafts, e.g. great saphenous vein (GSV), synthetic polytetrafluoroethylene (PTFE) grafts, or in some transplant centres, iliac artery allografts, have been used. The risk of contamination from gastrointestinal organisms means that natural tissue grafts are recommended to the use of synthetic grafts. Direct anastomosis was less commonly performed, but when possible, has shown successful results. This has been done either at the level of the stenotic CA (Aorta-CA anastomosis) in one patient, or more commonly at the level of PD arcade between the GDA and its branches (six patients), the middle colic artery (two patients), or the replaced right hepatic artery (one patient). The last case was a combined PD and right hepatectomy for cholangiocarcinoma, and the replaced right hepatic artery was anastomosed to the GDA to provide blood supply to the left hepatic artery (LHA) [20].

Endovascular stenting was performed either preoperative or postoperative in 14 patients, with good results [5,6,25-32]. However, in one patient, CAS was discovered after postoperative biliary and pancreatic leakage, needing two further laparotomies. The leakage eventually resolved with the help of endovascular angioplasty and stenting, and the patient survived after going through significant morbidity.

Endovascular dilatation without stenting was performed in one patient. Attempts at endovascular treatment failed in two patients due to failed CA cannulation. Open retrograde stenting during surgery is another useful technique that has been performed in three patients, with successful results in two.

Preservation of the collaterals supplying blood to the hepatic artery is another strategy utilised in 13 patients [14,17,19,24,33-41]. This usually involves the preservation of the pancreaticoduodenal arcade, which is formed of the GDA and its branches that communicate and receive blood from the IPDA from the SMA.

Complications

In this study, there were 10 (9 CAS, 1 SMA stenosis) significant complications related to coeliac or SMA stenosis (Table 4).

**Table 4:** Summary of complications in patients with coeliac artery stenosis IPMN: Intraductal Papillary Mucinous Neoplasm; HA: Hepatic Artery; MAL: Median Arcuate Ligament; PTFE: Polytetrafluoroethylene; POD: Postoperative Day; CAS: Coeliac Artery Stenosis.

Author, Year	Age, Sex	Pathology	Management	Outcome
Bong et al., 2007 [36]	56, M	IPMN, CAS	Reduced pulsation in HA, but no visible liver ischaemia. No treatment for CAS.	Gastric necrosis needed total gastrectomy, which was complicated by a leak. No mortality.
Farma et al., 2006 [5]	Unknown	CAS	Hepatic artery endarterectomy	Complicated by hepatic artery re-occlusion, delayed biliary anastomotic leak. No mortality.
Vellar et al., 1999 [25]	69, F	Ampullary, CAS	CAS discovered postoperatively after bile and pancreatic leak. Treated by endovascular CA stenting.	Leaking anastomoses needed two relaparotomies before stenting was performed, prolonged hospital stay. No mortality.
Peros et al., 2009 [8]	64, M	Pancreatic cancer, CAS	Aorto-hepatic bypass using PTFE graft, after CAS was discovered intraoperatively.	Stenosis at the vascular anastomosis, leading to hepatic ischaemia, needed endovascular stenting. Prolonged hospital stay. No mortality.
Sugae et al., 1990 [19]	61, M	Cholangiocarcinoma, CAS	CAS was diagnosed on preoperative angiography, intraoperative monitoring of hepatic perfusion. No additional procedures were performed.	Compromised vascularity of residual stomach, spleen, distal pancreas necessitated resection of these organs on POD 9 (Gastrectomy, total pancreatectomy, and splenectomy)
Lipska et al., 2009 [42]	50, M	Unknown, CAS	Diagnosed preoperatively. Hepatic artery pulse was present after removing the specimen, and the liver didn't show signs of ischaemia (steatotic), so no revascularisation was performed, but the patient died on POD2, and the autopsy showed liver ischemia.	The patient died suddenly on postoperative day 2. Autopsy confirmed severe liver ischaemia.
Schumacher et al., 2022 [43]	66, unknown	Pancreatic cancer, CAS	CAS was considered marginal. AoB preservation was impossible, GDA was ligated, pulse was found in proper hepatic artery.	Gastric, pancreatic and splenic ischemia, requiring re-laparotomy, gastrectomy, splenectomy, and resection of remnant pancreas.
Piardi et al., 2019 [12]	63, unknown	IPMN, CAS	Splenic artery aortic bypass (Splenic artery divided distally and anastomosed to the aorta).	Complicated by splenic abscess due to splenic devascularisation.
Aru et al., 2023 [44]	74, M	Pancreatic cancer, CAS	Intraoperative MAL division showed flow improvement on Doppler, but postoperative ischaemic hepatitis, necessitated relaparotomy and retrograde coeliac axis stenting.	Ischaemic hepatitis resolved after retrograde stenting.

In one patient, reduced pulsation was noticed in the GDA after clamping, however as the liver didn’t show signs of ischaemia (visually), no further action was taken. However, the patient became profoundly acidotic and had an emergency laparotomy on day 8, and a total gastrectomy with Roux-en-Y oesophagojejunostomy was performed, which was complicated by a leak. This patient survived but had an inpatient stay of four months [36].

In another patient, CAS was managed by hepatic artery endarterectomy, however, the HA occluded postoperatively leading to delayed bile leak, and hepatic abscess. This is the only case of endarterectomy in this review [5].

The importance of preoperative diagnosis, or adequate treatment after intraoperative identification is highlighted by a case reported by Vellar et al. [25], where the diagnosis was made postoperatively after biliary and pancreatic leakage needing two relaparotomies. Endovascular stenting was then performed, which allowed healing of the leaking anastomoses, and eventual discharge after significant morbidity.

One patient had aorto-hepatic bypass using PTFE, and was complicated by stenosis at the anastomosis, which resulted in hepatic ischaemia, and endovascular CA stenting. The patient survived but with a long hospital stay of 42 days.

Another patient was diagnosed with CAS preoperatively on a CT scan, but after specimen removal, pulse was still present in the hepatic artery, and no signs of liver ischaemia were observed in the cholestatic liver, so no further revascularisation procedures were performed. The patient died on postoperative day 2, and severe liver ischaemia was confirmed on autopsy. Although GDA clamp test is considered the gold standard and a more accurate method for detecting the need for revascularisation, this example gives credit to preoperative CT imaging as an accurate method of diagnosis and the need for treatment [42].

Underestimation of the degree of CAS can lead to significant morbidity, as evidenced in the case reported by Schumacher et al., where the CAS was considered marginal and no preoperative treatment was done. In this patient, the arc of Buhler (AoB) was also present, preservation was not possible due to close proximity to the tumour. Clamping AoB didn’t cause a reduction of the pulse in the proper hepatic artery, so no further action was taken. However, on postoperative day 9, the patient became unwell and the CT showed insufficient gastrojejunostomy, pancreaticogastrostomy, necrosis of the pancreatic remnant, and splenic artery thrombosis. Emergency laparotomy, total gastrectomy, splenectomy and resection of the pancreatic remnant were performed, and the patient was discharged after 44 days.

SMA Stenosis

SMA stenosis is much less commonly reported than CAS. In this review, only nine patients were reported. Table 5 shows a summary of these cases.

**Table 5:** Summary of patients with SMA stenosis, and management outcomes. POPF: Post-operative Pancreatic Fistula; MCA: Middle Colic Artery; IMA: Inferior Mesenteric Artery

Author	Age, sex	Diagnosis	Comments	Treatment of stenosis	Outcome
Sumi et al., 2000 [45]	69, F	Pancreatic cancer	Severe stenosis at the origin of SMA	Thrombectomy	Favorable
Tanigawa et al., 2004 [46]	58, M	Pancreatic cancer	Severe stenosis for 1 cm from the origin of the SMA	Preoperative endovascular stenting	Favorable
Gaujoux et al., 2009 [47]	63	Pancreatic cancer	-	Preoperative endovascular stenting	Complicated by grade C POPF
Gaujoux et al., 2009 [47]	68	Pancreatic cancer	-	Preoperative endovascular stenting	Favorable
Taniguchi et al., 2010 [48]	67, F	Pancreatic cancer	Retrograde blood flow through the MCA to the SMA	No extra procedures	Favorable
Kitaguchi et al., 2015 [49]	60, M	Pancreatic cancer	Occluded SMA, replaced CHA, blood flow was preserved after resection through the collateral arteries from CA to CHA to SMA	No extra procedures	Favorable
Tagkalos et al., 2018 [50]	64, F	Pancreatic cancer	SMA stenosis, missed on preoperative imaging, postoperative discovery, with elevated transaminases, failed angiographic recanalisation, successful treatment with Alprostadil and Anticoagulation.	Medical treatment with Alprostadil and Anticoagulation. IMA supplied collateral blood flow.	Favorable
Ohtsuka et al., 2019 [51]	73, M	Cholangiocarcinoma	Test clamp of GDA showed no change in intestinal color or pulsation in mesentery, so no extra procedures were performed.	No extra procedures. Later was found on CT that arc of Riolan supplied collateral blood flow from IMA to SMA.	Favorable
Gaujoux et al., 2009 [47]	Unknown	Unknown	Unrecognised SMA stenosis. 2 further relaparotomies and small bowel resections.	None	The patient died postoperatively due to bowel ischaemia.

Three patients had preoperative endovascular stenting with successful results [46,47]. In another three patients, collateral blood flow was supplied from the inferior mesenteric artery (IMA) through either the middle colic artery (MCA) or the arc of Riolan, therefore revascularisation was not performed [48,50,51]. One of these patients had concomitant CAS and SMA stenosis, and was discovered postoperatively with elevated transaminases. Endovascular treatment failed but collateral blood flow from the IMA was enough, and the patient was treated with Alprostadil.

One patient had replaced CHA from the stenosed SMA, therefore preservation of CHA and its collaterals that communicate with CA branches allowed preservation of blood supply to SMA, and thus no reconstruction was needed [49].

One patient had undetected SMA stenosis, which was discovered on postoperative day 1, and underwent two relaparotomies, small bowel resection, but died due to small bowel ischaemia [47].

Discussion

Whipple’s procedure is a technically demanding operation indicated for resection of malignant and premalignant conditions of the head of the pancreas, bile duct, and duodenum. Awareness of vascular anatomy, variations, and pathologic conditions affecting foregut and midgut arteries is crucial to safe surgical technique and good outcomes.

The operation almost always involves ligation and division of the gastroduodenal artery as well as the inferior pancreaticoduodenal artery (IPDA) which together with their branches form an arterial arcade around the head of the pancreas, and this arcade can form collateral blood supply in either direction in case of stenosis or occlusion of either the CA or the SMA.

If untreated, coeliac artery stenosis in a patient undergoing PD can lead to severe consequences, including increased risk of pancreatic or bile leakage, gastric and hepatic ischemia, and death [52,53]. Zhou et al. reported a significantly increased risk of biliary fistula after PD in CAS (27% vs 2.6%) [54]. In another study, coeliac artery stenosis was also found to be an independent risk factor for clinically relevant (grade B/C) post-operative pancreatic fistula, as well as increased risk of bile leak [55].

While severe stenosis requires intervention to maintain vascularity and prevent ischemic complications, it appears that there is no difference in outcome in those with <60% stenosis [19,53,55].

In this study, we reviewed the published literature on patients undergoing Whipple’s procedure with concomitant coeliac artery or superior mesenteric artery stenosis/or occlusion, and the different management strategies, and outcomes.

The prevalence of clinically relevant coeliac artery stenosis among patients undergoing pancreaticoduodenectomy is around 5% of cases [35,56]. Although higher prevalence rates have been reported, this is due to the inclusion of less clinically significant grades of stenosis. In a retrospective review of 989 patients by Al-Saeedi et al., 273 (27.5%) patients had CAS [55]. They classified patients according to the severity of the CAS into three groups - grade A (30-50% stenosis) in 17.9%, grade B (50-80%) in 8.4%, and severe CAS (>80%) in 1.3%. The rate of postoperative complications including liver failure and post-operative pancreatic fistula (POPF) was higher as the grade increased, but only reached statistical significance in severe CAS (>80%). Increase in average age of patients undergoing PD may also explain the increased rate of CAS in recent studies from previously reported due to the increased rate of atherosclerotic CAS in older patients [34].

Sugae et al. also classified CAS secondary to MALS, into types A, B and C according to degree and length of stenosis. They proposed a treatment algorithm according to the type of CAS; patients with type A don’t require any additional procedures, patients with type B require MAL division, while those with severe (type C) stenosis may require a revascularisation procedure in addition to MAL division [19].

MALS and atherosclerosis were the most common causes of CAS, while SMA stenosis is caused by atherosclerosis. In a study by Oikawa et al., independent predictors of atherosclerotic CAS are age older than 75 years, and ischaemic heart disease [34].

Before the advent of advanced computerised tomography technology, diagnosis was usually made either on catheter angiography preoperatively, intraoperatively after GDA clamping/division, or postoperatively due to ischaemic complications [23,57].

With modern imaging techniques, diagnosis is usually made preoperatively using dual or triple-phase computerised tomography scans. The accuracy of CT is variable in different studies. In a study by Yang et al. on 458 patients, CT had 97% accuracy, however, the sensitivity was only 58.3% [58]. Another series reported sensitivity of 96%, with overall accuracy of 92% [47]. Even with modern imaging, some cases were still diagnosed intraoperatively or postoperatively.

This highlights the importance of intraoperative confirmation of pulsatile flow in the hepatic artery after clamping of the gastroduodenal artery before ligation and division of the vessel. If pulse is reduced or not palpable, or evidence of liver/gastric ischemia is shown, a management strategy should be planned.

However, GDA clamping can also lead to false negative results and care should always be taken to interpret this in conjunction with the CT images [42].

Although GDA clamp test is mostly done to check blood supply in the hepatic artery, the pulsation in the small bowel mesentery should also be checked before irreversibly dividing the vessel, to avoid the dreadful outcome of mesenteric ischaemia and infarction.

CAS appearance on CT scans depends on the cause of the occlusion; in compression by MAL, a hooked appearance of the vessel is seen due to focal narrowing of the superior part of the CA by the MAL [58]. In atherosclerotic occlusion, narrowing, irregularity and calcification of the vessel wall is seen, as well as atherosclerotic changes in other arteries.

In a study on CT scans of 106 patients undergoing PD, Ito et al. found an increased number and size of collateral arteries within the meso-pancreas and around the pancreatic head in patients with moderate to severe CAS compared to non-CAS patients [59]. Gonen et al. reported a case of CAS diagnosed after a prominent GDA of 7mm was found on endoscopic ultrasound workup of pancreatic head mass, which was confirmed intraoperatively [40].

Management

Management of CAS or SMA stenosis in the context of pancreaticoduodenectomy depends on the cause, timing of diagnosis, and availability of technical expertise and skill. Preoperative diagnosis allows more time for planning and utilisation of resources to provide the best possible outcome.

If CAS stenosis is diagnosed preoperatively, there are two different approaches to management. The first is a two-stage procedure ensuring restoration of blood flow through the affected artery before contemplating on major resection. This can be achieved either by endovascular dilatation and stenting or MAL division which can be performed by laparoscopic or open approach [31]. The two-stage approach has the advantage of ensuring patency of CA prior to PD surgery, and avoids unpleasant surprises during surgery from failure of blood flow restoration, or prolonged surgery and resultant morbidity.

In one stage procedure, multidisciplinary team (MDT) discussion and proper planning are essential, as in more than 20% of patients, MAL division is not successful [60], and another approach may be necessary. Trial clamping of GDA should be done early during the procedure, and adequate blood flow should be restored before irreversible interruption of the vessel.

Endovascular dilatation and stenting of the coeliac artery is usually successful in restoring blood flow, however, there are cases where cannulation of the diseased artery is not possible [61,62], and thus another revascularisation procedure is necessary. Persistent or recurrent occlusion after stenting has been reported in the literature, but in patients undergoing the procedure for symptomatic MALS. In this study, there were no reports of re-occlusion after initial satisfactory results.

In the postoperative setting, endovascular stenting can be a very useful salvage procedure in case of ischaemia detected in the post-operative period, and can reduce the severity of ischaemic complications [25,27].

MAL division can be performed as a first-stage operation or during PD. The lesser sac is opened at the pars flaccida, then the oesophagus and stomach are retracted to the left. Dissection of the right crus of the diaphragm off the anterolateral border of the aorta followed by division of the abnormally inserted crural fibres, to clearly expose the origin of the coeliac artery [63].

MAL division alone is successful in most cases, however, in some patients, this fails to restore adequate blood flow necessitating further revascularisation procedure such as vascular reconstruction, on table endovascular stenting, retrograde stenting or preservation of collaterals.

Vascular reconstruction is an effective way of management of coeliac artery stenosis. Various techniques have been described, the most common being aorta to CHA bypass using autologous vein, iliac artery allografts or PTFE graft. Splenic artery has a long course and this has been utilised for revascularisation by detaching the distal end and anastomosis to the aorta or the SMA. This has shown successful results but was complicated in a study by splenic infarction and abscess. Direct anastomosis between the coeliac artery and aorta, IPDA and GDA, or GDA stump and middle colic artery has also been performed with good results.

The choice of a reconstruction technique depends on the patient's anatomy and surgeon's preference and experience. Preoperative thorough review of the vascular anatomy on an arterial phase CT is essential to accurately plan for the surgical strategy.

These techniques require necessary expertise and skills, and add to the operative time and the complexity of the operation which may not be suitable for some patients.

Preservation of the collaterals supplying blood to the hepatic artery is another effective strategy to preserve blood supply to the HA. However, the obvious limitation of this technique is the significant likelihood of the PD arcade being involved in malignant lesions of this area, even if not shown on preoperative imaging or on intraoperative assessment, and thus a higher incidence of positive oncological margins. This method is therefore only useful in benign pathology, or very early less aggressive tumours. Surgeons must be prepared in all cases for an alternative plan.

Arc of Buhler (AoB) is a rare anatomical variant which is a direct arterial communication between the CA and the SMA and is present in 1-3% of patients, and when preserved it can provide alternative blood flow to the hepatic artery in cases of CAS [64]. In this review, two patients had successful preservation of AOB and of blood supply to HA.

SMA stenosis is much less commonly encountered than CAS. In patients with SMA stenosis, preoperative stenting has been performed in several case reports with successful results. Unrecognised SMA may lead to small bowel ischaemia and infarction. However, in many cases, adequate collaterals from the IMA may be enough to allow resection without the need for revascularisation procedure.

Severe ischaemic complications result from either failure to identify, inadequate or delayed treatment after diagnosis of stenotic or occluded CA or SMA or both. Untreated CAS complications may manifest as a rise in liver enzymes signifying ischaemic hepatitis, necrosis of the stomach, or pancreatic remnant, with resulting biliary, pancreatic or gastric leakage. These complications lead to significant morbidity, length of stay, further operative procedures, and increased risk of mortality.

## Conclusions

This review highlights the importance of good quality and accurate interpretation of imaging in patients undergoing Whipple’s procedure. Unrecognised coeliac artery stenosis can result in a significant increase in POPF, liver ischaemia, bile leak, and death after surgery. SMA stenosis, albeit rare if not recognised, can prove fatal secondary to small bowel ischaemia. Intraoperative GDA clamp test and confirmation of adequate pulse in the hepatic artery and also the SB mesentery are crucial to avoid ischaemic complications.

Various technical methods have been utilised with varying success in order to prevent the consequences of CAS or SMA stenosis. There is no evidence that one approach has an advantage over another, and surgeons should seek the technique that is most feasible and suited to the local expertise and skill, without impacting patient safety or the purpose of this operation.
